# Unveiling the hidden regulators: how post-translational modifications influence the progression and treatment of hepatocellular carcinoma

**DOI:** 10.3389/fonc.2026.1726574

**Published:** 2026-02-25

**Authors:** Shuaiyong Qi, Xiang Yong, Zhixian Ding, Mengxue Hu, Yafen Li, Lili Li, Huaiyuan Hu, Heng Tang

**Affiliations:** 1 Central Laboratory, Wanbei Coal Electric Group General Hospital, Suzhou, China; 2 Key Laboratory of Tumor Pathology, Wanbei Coal Electric Group General Hospital, Suzhou, China

**Keywords:** clinical translation, hepatocellular carcinoma, metabolic reprogramming, post-translational modifications, therapy

## Abstract

Hepatocellular carcinoma (HCC) is a common and aggressive primary liver cancer. Due to its high incidence and fatality rates, it poses a serious threat to global public health. Protein post-translational modifications (PTMs) are crucial regulatory mechanisms that occur after translation and fine-tune cellular functions. Common PTM types-including phosphorylation, ubiquitination, acetylation, methylation, glycosylation, ubiquitin-like modifications (such as UFMylation and SUMOylation), and Lactylation-affect protein activity, stability, subcellular localization, and interaction networks. These modifications dynamically regulate various biological processes in response to internal and external stimuli. Dysregulated PTMs have been intimately associated with the development, spread, and resistance to treatment of HCC in the setting of cancer. This review provides new insights into the molecular mechanisms underlying HCC by systematically examining the roles of PTMs. It also seeks to inform therapeutic strategies and improve diagnosis and prognostic assessment.

## 1 Introduction

**Figure 1:**
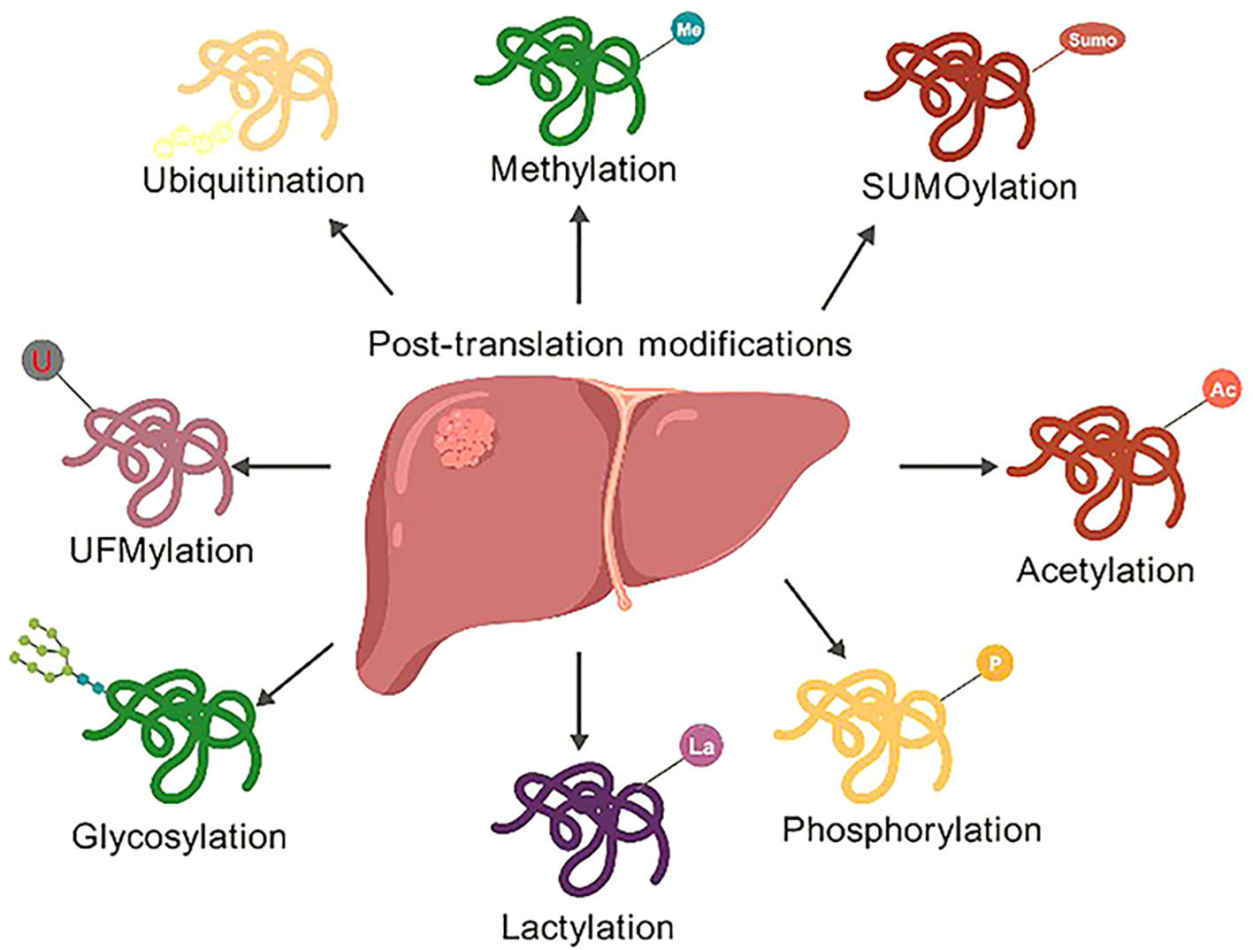
Schematic representation of PTMs in the liver. The target organ is represented by the central pink liver emblem, while several PTM channels are indicated by radiating arrows. The following are important changes: lactylation, glycosylation, phosphorylation, acetylation, ubiquitination, methylation, SUMOylation, and UFMylation. To show their many functions in hepatic protein regulation, each PTM is represented as a colored wave pattern with functional groups annotated.

After protein synthesis, PTM is a crucial regulatory process that dynamically alters protein subcellular localization, stability, and function by cleaving structures or covalently attaching chemical groups (1–3). It serves as a critical regulatory mechanism regulatory mechanism in living organisms, tremendously expanding the diversity and dynamics of protein functions. PTM is a key regulatory mechanism that precisely modulates protein function, stability, and subcellular localization via covalent addition of chemical groups or enzymatic cleavage of specific bonds (**Figure 1**) (4). In recent years, the regulatory roles of novel PTMs such as lactylation and UFMylation in HCC have been increasingly revealed. For instance, in HCC, histone lactylation promotes the Warburg effect by activating glycolysis-related genes such as HK2 and LDHA (5). Conversely, UFMylation deficiency results in exacerbated endoplasmic reticulum stress as well as resistance to ferroptosis (6–8). This review is the first to systematically integrate the interactive networks of these emerging PTMs with conventional modifications and proposes the concept of a “PTM code”, offering a novel framework for the precise subtyping of HCC. Dysregulation of PTMs is strongly associated with various diseases, such as cancer, metabolic syndrome, and neurodegenerative disorders (9–12).

According to the WHO International Agency for Research on Cancer’s most recent 2022 data (13), Asia accounts for 58.3% of all liver cancer cases worldwide. Specifically, 49.2% of cases and 56.1% of fatalities occur in this region. Primary liver cancer frequently appears subtly; more than 85% of patients are diagnosed at a stage involving some degree of liver failure or with tumors adjacent to major blood vessels, which means they miss the best opportunity for surgery. The most prevalent type of primary liver cancer, HCC, usually develops in association with chronic liver disease. In patients with HCC, it is an aggressive cancer that is closely linked to a poor prognosis (14). Ninety percent of cases of liver cancer are HCC, which has a significant molecular heterogeneity (15). While advanced patients require systemic chemotherapy and immunotherapy, early-stage HCC can be treated with surgery or ablation (16–18). The “PTM code” in HCC constitutes a multi-layered, dynamic, and complex system. Its core components primarily encompass a variety of modification types, such as phosphorylation, acetylation, ubiquitination, methylation, glycosylation, lactylation, SUMOylation, and succinylation, among others. These modifications do not exist in isolation; instead, they act on precise amino acid residue sites of specific proteins, forming distinct modification site maps (19). More crucially, different types of PTMs at various sites on the same protein can coexist and interact, forming complex combinatorial modification patterns. In HCC, the functions of key signaling nodes are precisely regulated by this intricate “PTM code,” where the combination and sequence of these modifications determine cellular fate decisions. For example, the tumor suppressor protein p53 serves as a paradigm of PTM regulation. Under stress conditions such as DNA damage, ATM/ATR kinase-mediated phosphorylation at Ser15 serves as the initiating signal for p53 activation. Subsequently, histone acetyltransferase-mediated acetylation at sites like Lys382 significantly enhances p53’s DNA-binding capacity, promoting target gene transcription (20). Concurrently, ubiquitination mediated by the E3 ubiquitin ligase MDM2 targets p53 for degradation, forming a negative feedback loop. The specific combination and spatiotemporal order of these phosphorylation, acetylation, and ubiquitination modifications constitute a precise “instructional code” that determines whether a cell undergoes cell cycle arrest, apoptosis, or senescence. Furthermore, mutant forms of p53 not only lose normal function but may also acquire aberrant “PTM codes,” thereby gaining oncogenic functions that promote tumor growth (21).

In summary, deciphering the “PTM code” on core signaling proteins is crucial for gaining a deeper understanding of HCC pathogenesis, revealing tumor heterogeneity, and identifying new therapeutic targets. Future research needs to integrate high-throughput proteomics, functional validation, and computational modeling to comprehensively decode the complex PTM regulatory network in HCC.

## 2 Phosphorylation and HCC

Kinases catalyze phosphorylation, a post-translational modification that phosphatases can reverse. Driven by energy from ATP hydrolysis, phosphorylation is characterized by the covalent attachment of a phosphate group (-PO_4_³^-^) to particular amino acid side chains of target proteins (22). In eukaryotes, the hydroxyl groups of serine (Ser), threonine (Thr), and tyrosine (Tyr) residues serve as the primary phosphorylation sites. This modification results in the formation of phosphoester linkages (23). Phosphorylation is a key regulatory mechanism that affects gene expression, signal transmission, cell division, and protein interactions. As such, it is essential to cellular processes. Mutations at critical phosphorylation sites can promote carcinogenesis and malignant development by encouraging aberrant proliferation, invasion, metastasis, and inhibiting apoptosis in cancer cells (22, 24, 25).

**Figure 2:**
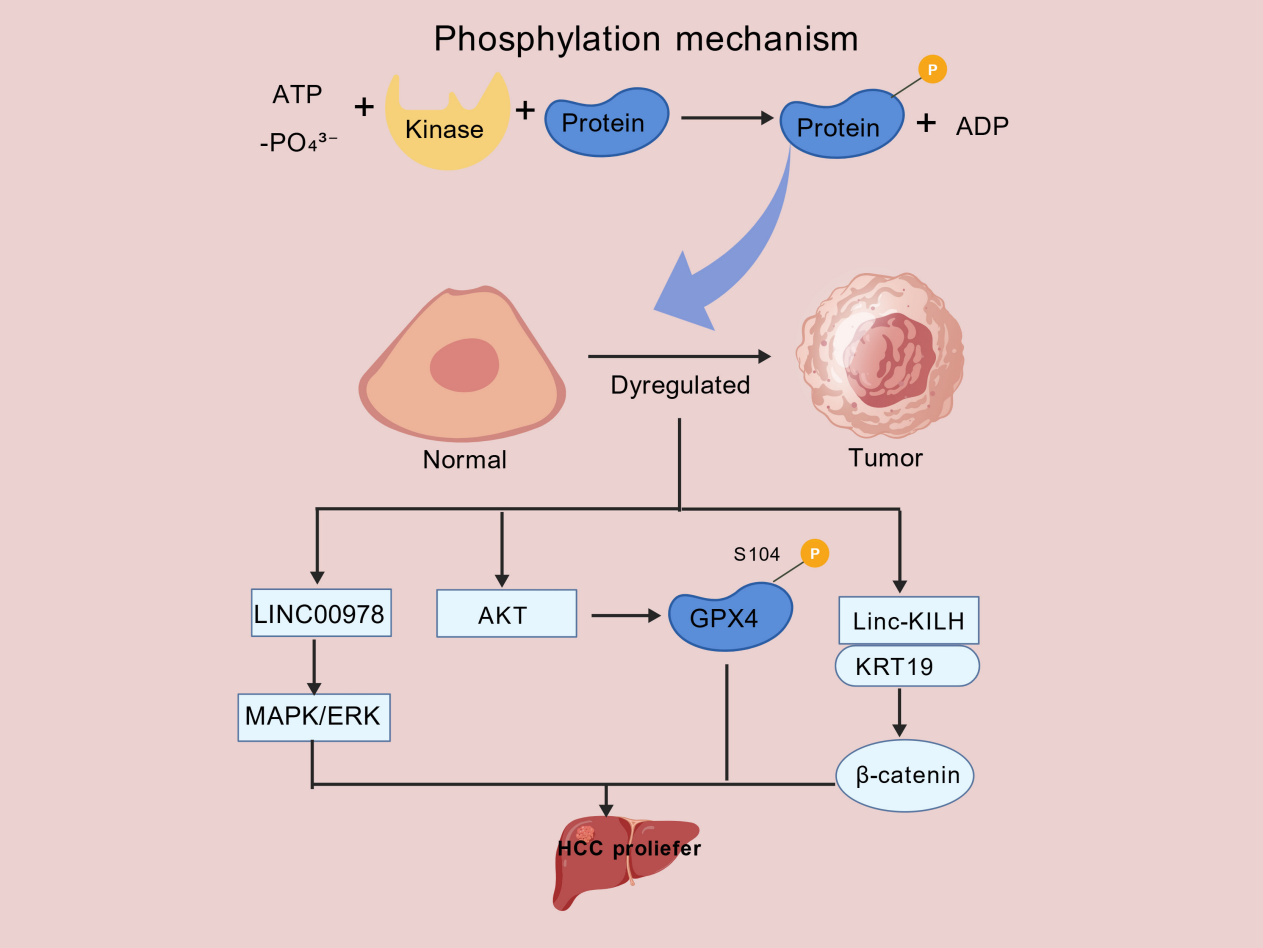
Dysregulated phosphorylation in HCC progression. The figure summarizes the pivotal role of protein phosphorylation in HCC pathogenesis. Under physiological conditions, kinases catalyze the transfer of a phosphate group (-PO_4_³^-^) from ATP to specific residues on substrate proteins, a reversible process regulated by phosphatases to maintain homeostasis. In HCC, this dynamic equilibrium is disrupted, leading to constitutive activation of oncogenic signaling pathways that drive malignant proliferation. Examples highlighted in this figure include: the LINC00978/MAPK/ERK axis promoting proliferation. the AKT/CKB/GPX4 axis, where AKT-mediated CKB phosphorylation (T133) leads to subsequent GPX4 phosphorylation (S104) and ferroptosis resistance and the Linc−KILH/KRT19/β−catenin axis, in which Linc−KILH inhibits KRT19 phosphorylation to facilitate β−catenin activation. Collectively, these dysregulated phosphorylation events converge to promote tumor progression, underscoring their potential as therapeutic targets.

Mutations at critical phosphorylation sites can promote carcinogenesis and malignant development by encouraging aberrant proliferation, invasion, metastasis, and by inhibiting apoptosis in cancer cells. This process promotes cell proliferation, metabolic reprogramming, and metastasis. Zhang et al. discovered that LINC00978 is closely linked to a poor patient prognosis and is markedly elevated in HCC tissues. The MAPK/ERK pathway’s activation is a contributing factor in its tumor-promoting mechanism, which promotes HCC cell proliferation, cell cycle progression, and survival (26). Wu et al. also showed that a receptor protein activates the non-canonical protein kinase function of creatine kinase B (CKB) via mediating T133 phosphorylation of CKB via AKT. Glutathione peroxidase 4 (GPX4) is further phosphorylated at S104 by phosphorylated CKB, which prevents HSC70 binding and chaperone-mediated autophagy. This stabilizes GPX4 and prevents ferroptosis. Clinical investigations showed that in HCC tissues, simultaneous phosphorylation at CKB T133 and GPX4 S104 correlates positively with GPX4 protein expression and strongly associates with adverse patient outcomes (27). The long non-coding RNA Linc-KILH, on the other hand, is abundantly expressed in HCC and substantially associated with a poor prognosis, according to Zhang et al. By binding directly to keratin 19 (KRT19), Linc-KILH stimulates membrane translocation of KRT19, suppresses its phosphorylation at Ser35, and improves its interaction with β-catenin. This promotes β-catenin’s nuclear translocation and oncogenic signaling pathway activation (28). However, from a metabolic standpoint, Davide et al. demonstrated the involvement of LPAR6 in liver cancer. By altering energy metabolism, inhibiting oxidative phosphorylation, and increasing lactate fermentation, this gene fosters a phenotype resistant to chemotherapy. Functional tests revealed that whereas LPAR6 knockdown restores drug sensitivity in tumor cells, its overexpression causes chemoresistance (29). Together, these studies demonstrate that abnormal phosphorylation events play a key role in HCC pathophysiology and are closely linked to malignant traits, including drug resistance, metabolic reprogramming, metastasis, proliferation, and ferroptosis resistance. Additionally, they draw attention to the possibility of phosphorylation indicators as biomarkers for prognosis. More significantly, these results highlight the practical importance of focusing on particular phosphorylation nodes (kinases, phosphatases, or substrate phosphorylation sites) and disrupting phosphorylation-dependent protein interactions in order to improve prognostic assessment, overcome drug resistance, and perform precision diagnosis in HCC (**Figure 2**). A crucial theoretical underpinning and possible therapeutic targets for creating next-generation tailored treatment approaches will be provided by methodically figuring out the phosphorylation regulatory network unique to HCC. Comprehensive studies of the phosphorylation regulatory network in HCC will open up new avenues for the development of precise treatment and diagnostic approaches.

## 3 Ubiquitination and HCC

Ubiquitin (Ub), a small protein, exists in multiple compartments of eukaryotic cells. It is made up of 76 amino acids and has a molecular weight of about 8.5 kDa. The ubiquitination process involves a highly regulated three-step enzymatic cascade. First, a ubiquitin-activating enzyme (E1) activates ubiquitin in an ATP-dependent manner. Next, ubiquitin is transferred to a ubiquitin-conjugating enzyme (E2). Finally, a ubiquitin ligase (E3) recognizes specific substrate proteins and catalyzes the covalent attachment of ubiquitin’s C-terminal glycine to the ϵ-amino group of a lysine residue on the substrate (30). Deubiquitinating enzymes (DUBs) selectively hydrolyze the peptide or isopeptide bond linking ubiquitin to substrates, thereby removing the ubiquitin tag and reversing its regulatory effects. This process is reversible (31). Ubiquitination plays crucial regulatory roles in various cellular processes, including protein degradation, apoptosis, epigenetic control, DNA damage response, autophagy, and immune reactions. Recent research continues to uncover its diverse functions (32–34). By these means, ubiquitination affects cellular communication and homeostasis in general as well as protein stability, location, and function. Dysregulation of ubiquitination is closely linked to the pathophysiology of many human diseases, including cancer, autoimmune disorders, inflammatory conditions, neurodegenerative diseases, and metabolic syndromes. This highlights ubiquitination’s potential as both a biomarker and a therapeutic target.

**Figure 3:**
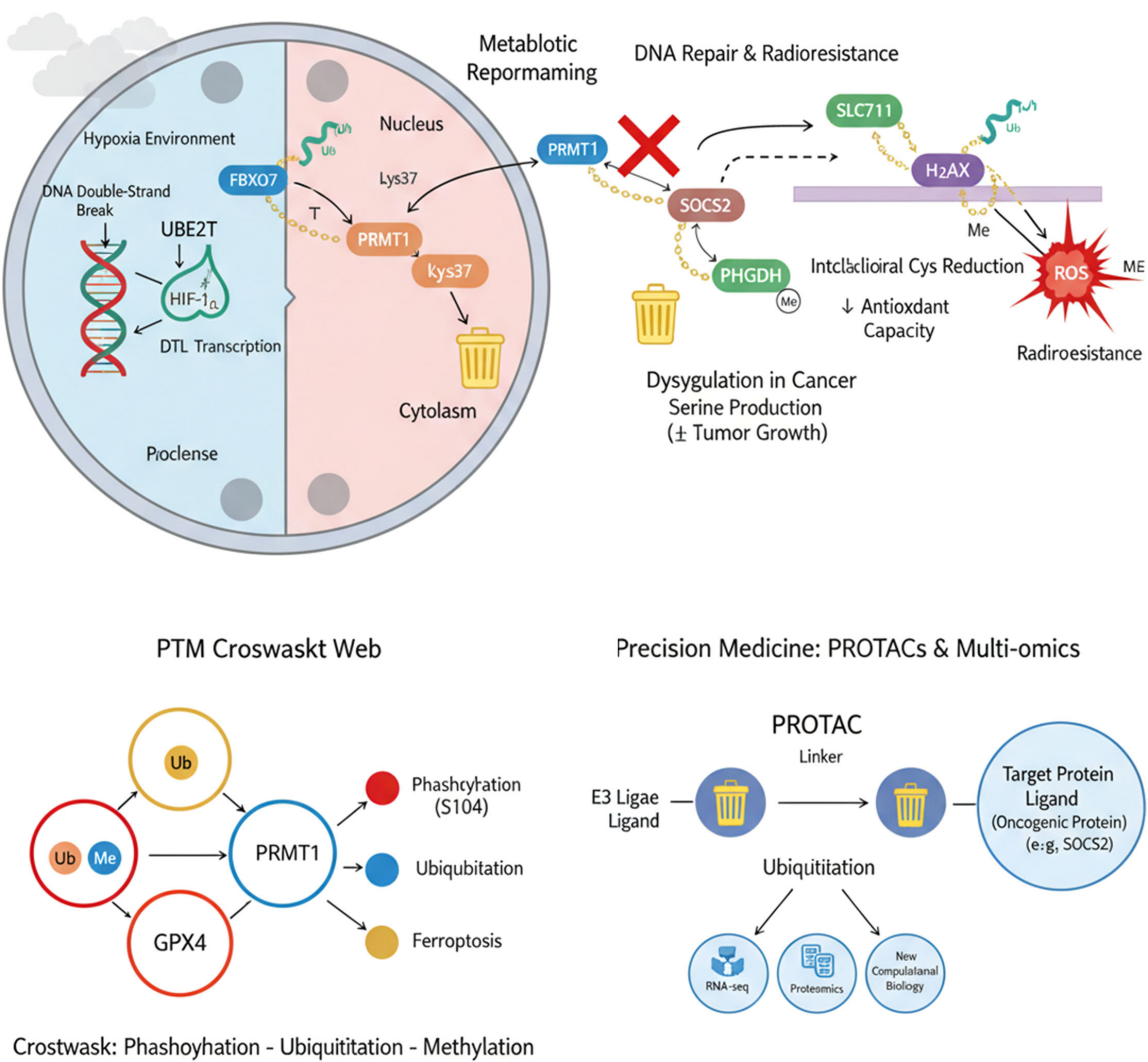
Compartmentalized ubiquitination network and functional integration in HCC. In the cytoplasm, the E3 ubiquitin ligase FBXO7 mediates ubiquitination−dependent degradation of PRMT1 at lysine 37 (K37), thereby suppressing the PHGDH−dependent serine synthesis pathway and restricting tumor growth. Concurrently, SOCS2 promotes ubiquitination−mediated degradation of SLC7A11, inducing ferroptosis and enhancing radiosensitivity of HCC cells. In the nucleus, hypoxia−induced HIF−1α transcriptionally activates the E3 ligase DTL, enabling its involvement in hypoxic stress response. Under radiation stress, the ubiquitin−conjugating enzyme UBE2T facilitates ubiquitination of histone H2AX, promoting DNA damage repair and conferring radioresistance. These findings collectively illustrate that the ubiquitination network operates through precise compartmentalized regulation and plays key roles in HCC progression. Future research should further elucidate the crosstalk between ubiquitination and other post−translational modifications (e.g., phosphorylation, as exemplified by GPX4 regulation), as well as explore multi−omics−based biomarker discovery and novel targeting strategies—such as PROTAC−mediated protein degradation—for potential applications in precision therapy of liver cancer.

Serine production is frequently necessary for the proliferation of cancer cells. According to Luo et al. (35), Protein arginine methyltransferase 1 (PRMT1) is increased in HCC. It methylates and activates phosphoglycerate dehydrogenase (PHGDH), thereby promoting serine production. The E3 ligase FBXO7 interacts with PRMT1 and causes its ubiquitination at lysine 37, which results in proteasomal degradation and ultimately suppresses serine production in HCC, the same researchers found in their investigation of the molecular underpinnings of HCC (36). Notably, Chen et al. showed for the first time that the denticleless E3 ubiquitin protein ligase homolog (DTL) is a downstream effector of HIF-1α and is transcriptionally activated by HIF-1α under hypoxic settings (37). One of the main reasons why radiation fails in HCC is radioresistance. According to Sun et al., ubiquitin-conjugating enzyme E2T (UBE2T) is upregulated in HCC tissues and facilitates cell cycle progression by mediating ubiquitination of H_2_AX/γH_2_AX, thereby promoting DNA repair and radioresistance (38). Chen et al. identified suppressor of cytokine signaling 2 (SOCS2) as a potential molecular marker in HCC through RNA-seq and bioinformatic analysis. SOCS2 promotes ferroptosis by facilitating ubiquitination and degradation of SLC7A11. This suggests that targeting SOCS2 could enhance radiation therapy effectiveness and improve patient outcomes (39). A thorough examination of the ubiquitination regulatory network unique to liver cancer will offer a crucial scientific foundation for creating tailored treatments and ubiquitination-based diagnostic tools, ultimately promoting the development of precision medicine paradigms for HCC. Future research should focus on the complex interactions between phosphorylation and ubiquitination networks in HCC (**Figure 3**).

Although existing studies have preliminarily revealed the critical role of ubiquitination in HCC metabolism, stress adaptation, and therapeutic resistance, significant limitations remain in our understanding of this field. There is an urgent need to shift from isolated case descriptions to a systematic, dynamic, and integrative analytical paradigm. Current research predominantly focuses on linear relationships between “single E3/E2, single substrate, and single phenotype”—for example, the FBXO7, PRMT1, and serine synthesis axis or the UBE2T, H_2_AX, and radioresistance axis—which fail to reveal the overall topology, dynamic hierarchy, and complex cross-talk of the ubiquitination modification network in HCC with other PTMs such as phosphorylation and methylation. For instance, the activity of PRMT1 is regulated by both methylation and ubiquitination. It remains unclear how these two modifications cooperate or antagonize each other. The stability of GPX4 is regulated by phosphorylation at serine 104 (S104) and is inevitably under the surveillance of the ubiquitination network. How these two types of modifications integrate to determine the cell’s ferroptosis fate is still unknown. The lack of systematic analysis of such multi-dimensional regulatory networks—encompassing various modifications, interactions, and temporal dynamics - is a major shortcoming in the study of ubiquitination in HCC.

Only through such system-level, mechanism-integrated research and by closely linking discoveries with clinical challenges can knowledge of ubiquitination ultimately be effectively translated into precise diagnosis and treatment strategies for liver cancer.

## 4 Acetylation and HCC

Protein acetylation is a critical post-translational modification mechanism that dynamically controls protein function and activity. It does so by covalently adding acetyl groups (-COCH_3_) to lysine residues. Lysine acetyltransferases (KATs) catalyze this process, whereas lysine deacetylases (KDACs) reverse it, which creates a highly dynamic and reversible regulatory network (40–43). Acetylation alterations in HCC play a significant role in important pathological processes such metabolic reprogramming, signal transmission, and tumor immune microenvironment remodeling, in addition to their conventional function in epigenetic control. In addition to being a major metabolic node that integrates the metabolic pathways for glucose, lipids, and amino acids, acetyl-CoA is a direct donor of acetyl groups. It also acts as a crucial molecular link between metabolic status and epigenetic regulation by regulating the acetylation levels of both histone and non-histone proteins (44, 45).

One reliable biomarker for the clinical diagnosis of HCC is alpha-fetoprotein (AFP). According to Xue et al., the acetyltransferase CBP and the deacetylase SIRT1 co-regulate the acetylation of AFP. Acetylation at lysine residues K194, K211, and K242 increases AFP’s oncogenic activity. It also considerably lengthens its half-life and prevents its destruction through ubiquitination. Acetylation promotes the interaction of AFP with PTEN and caspase-3, thereby inhibiting the tumor-suppressive function of PTEN and apoptosis mediated by caspase-3. The PI3K/AKT signaling pathway is subsequently activated, which improves HCC cells’ capacity for invasion, migration, and proliferation (46). In addition to AFP, other proteins’ acetylation changes are also important for carcinogenesis. According to Ye et al, the STAT3 signaling pathway is activated when the G protein α subunit (GαS) is acetylated at K28, which causes it to move from the plasma membrane to the cytoplasm (47). Wang et al. showed that acetylation regulates PHGDH, a protein markedly elevated in human breast cancer, potentially linked to decreased RNF5 expression. This acetylation alters PHGDH’s catalytic activity, promoting serine synthesis to support cancer cell proliferation (48).

**Figure 4:**
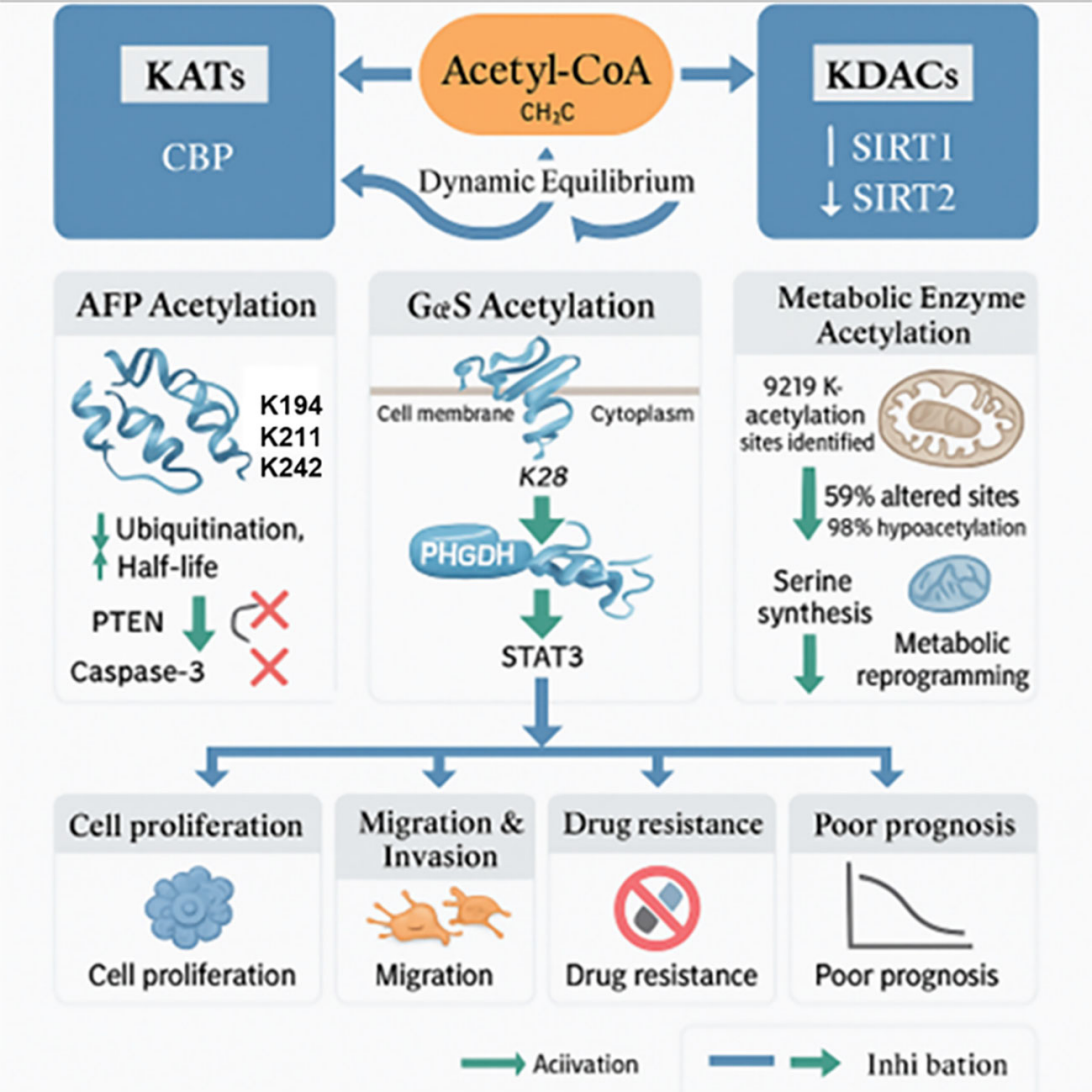
Functional roles of protein acetylation in cellular regulation and disease progression. In HCC, the equilibrium between acetylation (mediated by KATs such as CBP) and deacetylation (catalyzed by KDACs including SIRT1/SIRT2) is tilted toward hypoacetylation, largely due to KDAC overexpression. This imbalance promotes tumor progression through several key pathways (1): AFP acetylation at specific lysine residues enhances its stability, facilitates interaction with PTEN and caspase−3, and activates the PI3K/AKT axis, thereby driving proliferation, migration, and invasion (2); GαS acetylation triggers its cytoplasmic translocation and activates STAT3 signaling, which promotes cell migration; and (3) broad hypoacetylation of metabolic enzymes such as PHGDH—linked to SIRT2 overexpression—reprograms metabolism to sustain tumor growth. Together, these dysregulated acetylation events foster malignant phenotypes including sustained proliferation, invasion, metabolic adaptation, therapy resistance, and poor prognosis, underscoring protein acetylation as a central regulatory layer and a promising source of therapeutic targets in HCC.

The acetylation of metabolic enzymes is significantly dysregulated in HCC. Quantitative acetylomic analysis revealed that 98% of acetylated sites in HCC tissues exhibit hypoacetylation. Additionally, 59% of these sites show significant changes. These alterations are mainly found in metabolic enzymes located in the cytoplasm and mitochondria. Lu et al. from the Chinese PLA General Hospital identified 9219 lysine acetylation sites in HCC samples from the Chinese PLA General Hospital. They also discovered that pathways such as fatty acid metabolism, carbon metabolism, and amino acid biosynthesis showed significantly reduced acetylation of metabolic enzymes. These pathways are represented by enzymes including mitochondrial acyl-CoA dehydrogenases and pyruvate dehydrogenase (49). SIRT2 overexpression is closely associated with this extensive hypoacetylation. Furthermore, cellular models have demonstrated that SIRT2 overexpression inhibits glycolysis and oxidative phosphorylation, thereby reshaping cellular metabolism to promote rapid tumor growth (49). SIRT1, a crucial deacetylase, is also overexpressed in HCC and strongly promotes carcinogenesis. Central South University research by Cao’s group showed that SIRT1 causes RANBP2-mediated SUMOylation of FTO, which results in its destruction. This ultimately promotes the progression of HCC by increasing the m^6^A modification level of tumor suppressor genes such as GNAO1. In conclusion, the initiation, development, metastasis, and medication resistance of HCC are all significantly regulated by protein acetylation (**Figure 4**). A poor patient prognosis is directly associated with the abnormal expression of acetylation-related proteins. With expanding knowledge of the acetylation regulatory network and improvements in tailored intervention technologies, new methods to modify acetylation changes may provide treatment options for patients with HCC. Acetylation in HCC regulates important protein activities, metabolic reprogramming, and epigenetic pathways, all of which have a significant impact on tumor development and progression. Several important questions remain unanswered in this field. Therefore, future studies should focus on the following areas. First, to improve molecular categorization, subtype-specific acetylation landscapes and their relationships with clinicopathological characteristics must be systematically clarified. Second, more research should focus on non-histone acetylation. This includes studying the dynamic regulatory processes underlying signal transduction and discovering new substrates beyond metabolic enzymes. Third, it is essential to develop highly selective KATs/KDACs inhibitors and evaluate their effectiveness alongside immune checkpoint inhibitors and existing targeted therapies. Fourth, to understand the cooperative regulatory networks from a systems biology standpoint, more focus should be placed on the interactions between acetylation and other post-translational modifications, including phosphorylation, ubiquitination, and methylation. Lastly, initiatives supporting the clinical application of acetylation-based biomarkers for liquid biopsy and related regulatory agents are crucial to transform fundamental research into practical diagnostic and treatment methods. Given continuous advancements in proteomics, chemical biology, and gene-editing technologies, a better understanding of the acetylation regulatory network is anticipated. This may open new opportunities for precision medicine in HCC.

## 5 Methylation and HCC

Methylation, a fundamental process of epigenetic control, affects the development, progression, and treatment resistance of HCC (50, 51). Tumor suppressor genes are silenced or proto-oncogenes are activated as a result of DNA methylation, which is mediated by DNA methyltransferases (DNMTs) and includes the addition of methyl groups to cytosine inside CpG islands (52). For instance, global hypomethylation promotes genomic instability and transposon activation, whereas promoter hypermethylation of tumor suppressor genes such as RASSF1A, APC, GSTP1, and p16 frequently occurs in HCC (53–56). Methylation of the MGMT gene is strongly associated with shorter disease-free survival in patients, indicating that abnormal methylation patterns could serve as early diagnostic markers. Furthermore, histone methylation dynamically regulates chromatin structure through methyltransferases (such as EZH2-mediated H3K27me3) and demethylases (such as KDM4B/KDM6B), affecting migration, proliferation, and apoptosis resistance of liver cancer cells (57, 58). Overexpression of EZH2 accelerates the development of HCC by suppressing tumor suppressor genes. In contrast, KDM5C/JARID1B’s demethylation activity opens chromatin, allowing the expression of genes linked to metastasis. PRMT9 is closely linked to patient survival and is highly expressed in HCC. PRMT9 is highly expressed in HCC and its overexpression is closely associated with Hepatitis B viral antigens (59). PRMT9-mediated methylation activates the SLC7A11/GPX4 axis and enhances antioxidant defense by upregulating CD44 expression through HSPA8-mediated transcriptome reprogramming (60).

**Figure 5:**
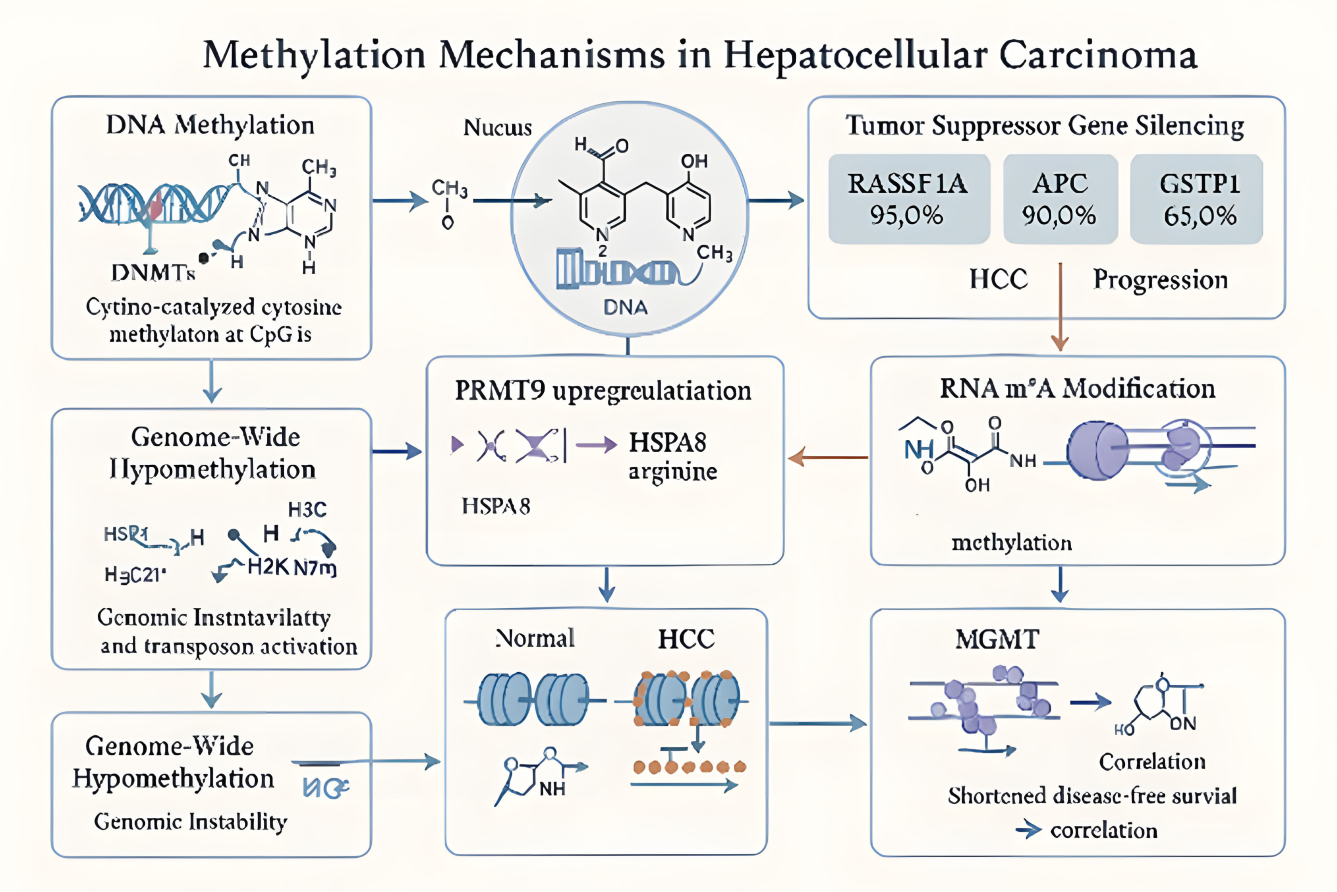
Dysregulation of methylation networks in HCC. The coordinated dysregulation of DNA hypermethylation at specific loci, genome-wide DNA hypomethylation, and protein arginine methylation-driven RNA modifications creates a pro-tumorigenic epigenetic landscape that silences tumor suppressors, induces genomic instability, rewires gene expression, and correlates with adverse clinical outcomes in HCC.

RNA methylation, such as m^6^A modification, is another important factor in HCC. The m^6^A methyltransferase complex component KIAA1429 is guided by the long non-coding RNA GATA3-AS to methylate specific sites on GATA3 pre-mRNA. This methylation reduces RNA stability and promotes HCC cell proliferation and metastasis (61). Therapeutic approaches targeting methylation modifications show encouraging potential. For example, 5-aza-2′-deoxycytidine (5-Aza-CdR), a methyltransferase inhibitor, can reverse the silencing of tumor suppressor genes; additionally, inhibiting EZH2 or KDM family members may offer new strategies to prevent HCC metastases (62). In conclusion, abnormal methylation changes have multifaceted regulatory consequences on HCC molecular networks, offering crucial information for targeted treatment, prognostic assessment, and early diagnosis. Nevertheless, important issues and unmet research needs remain. Future research should concentrate on clarifying the spatiotemporal variability of methylation patterns among various subtypes of HCC and how they dynamically change as the disease progresses and undergoes treatment. A viable avenue is the creation of more effective and specific inhibitors that target important methylation regulators including DNMTs, EZH2, and PRMTs. These inhibitors should be rationally combined with other treatment approaches such as immunotherapy and targeted therapy. A more thorough understanding of the epigenetic landscape in HCC will also be possible by investigating the interactions between methylation and other epigenetic alterations as well as metabolic pathways (**Figure 5**). Strong validation in sizable cohorts and well planned clinical trials are necessary for the clinical translation of methylation-based biomarkers and targeted treatments. By addressing these factors, we can better understand the epigenetic processes underlying HCC. This understanding will open the door to new approaches for diagnosis and treatment.

## 6 Glycosylation and HCC

Glycosylation is an important form of post-translational modification that plays a central regulatory role in the initiation, progression, and metastasis of HCC. This modification process is catalyzed by a series of glycosyltransferases located in the endoplasmic reticulum and Golgi apparatus. It uses nucleotide sugars as glycosyl donors to covalently attach glycan chains to specific amino acid residues, such as asparagine and serine/threonine, on proteins or lipids.

**Figure 6:**
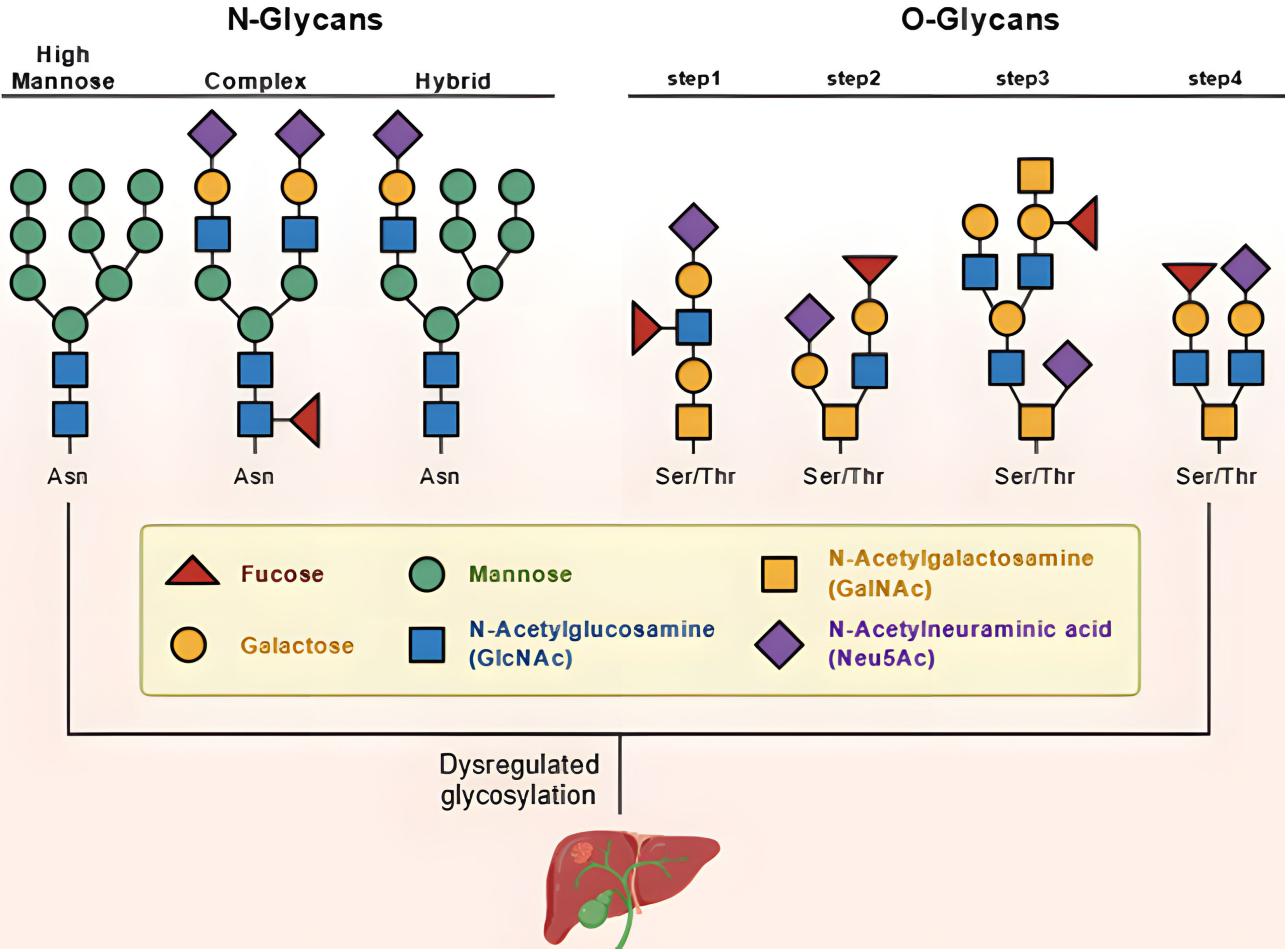
Structural diversity of N- and O-glycans and their dysregulation in HCC. This figure depicts the biosynthesis of N-linked and O-linked glycans and their aberrant regulation in pathologies such as cancer. N-glycans are attached to Asn residues and mature from high-mannose precursors to complex or hybrid structures through sequential enzymatic steps in the endoplasmic reticulum and Golgi apparatus. O-glycans are initiated on Thr or Ser residues, beginning typically with N-acetylgalactosamine (GalNAc) and extended by additional sugars. Both pathways utilize a defined set of monosaccharides, including mannose, galactose, N-acetylglucosamine (GlcNAc), fucose, and sialic acid (N-acetylneuraminic acid, Neu5Ac), whose arrangement determines glycan function. The label “Dysregulated glycosylation” highlights that altered glycan synthesis—such as increased branching, fucosylation, or sialylation—is a hallmark of cancers like HCC. These changes modulate protein stability, cell-cell adhesion, receptor signaling, and immune interactions, contributing to tumor progression, metastasis, and therapy resistance. Thus, glycan alterations serve as potential diagnostic biomarkers and therapeutic targets in oncology.

Through the stepwise addition of monosaccharide units, precise glycan structures are assembled (63). Glycosylation critically influences protein folding, stability, subcellular localization, and molecular recognition, thus modulating biological functions and cellular signaling (61, 62, 64, 65). It is reported that the majority of glycosylated serum proteins are synthesized by the liver, including acute-phase proteins (APPs). Altered glycosylation patterns are observed in all major liver diseases, highlighting their potential role in disease pathogenesis (66). Physiological disturbances such as hepatitis and neoplastic transformation trigger the acute-phase response and the release of inflammatory cytokines. These cytokines modify the glycosylation machinery in hepatocytes and alter the expression of glycosyltransferases, leading to further changes in APP glycosylation. N-linked glycosylation (N-glycosylation) modifies proteins at asparagine (Asn) residues, regulating their folding and stability. In HCC, core fucosylation catalyzed by FUT8 is significantly elevated, leading to increased levels of the serum biomarker AFP-L3, which exhibits over 90% specificity and serves as a key indicator for early diagnosis (67). Fucosylation of FOLR1 enhances folate uptake. It also induces upregulation of E-cadherin and downregulation of N-cadherin, which drives tumor cell metastasis (68). Research by Lyu et al. in Redox Biology revealed that in HCC, MerTK forms homo- and heterodimers through N-glycosylation at Asn294 and Asn454. This modification enhances MerTK’s stability and drives metabolic reprogramming (69). Zhou et al. demonstrated in multiple mouse models that high fructose promotes HCC progression. They also showed that acetate, a gut microbiota-derived metabolite of fructose, enhances O-GlcNAcylation to facilitate HCC proliferation. They further elucidated a molecular mechanism by which acetate - a gut microbiota-derived metabolite of fructose - enhances O-GlcNAcylation to facilitate HCC proliferation. This study provides an important theoretical basis and novel perspectives for the rational use of added sugars and the prevention of metabolic diseases (70). Fang et al. revealed that advanced glycation end products (AGEs), which accumulate in the extracellular matrix of fatty liver/diabetic livers, promote structural changes in liver collagen and increase ECM viscoelasticity, thereby facilitating HCC initiation through activation of the YAP pathway. This mechanistic insight offers new strategies for the prevention, diagnosis, and treatment of HCC associated with fatty liver and diabetes (71). Although current research has established the critical role of glycosylation in the development and progression of HCC, significant limitations remain in the systematic understanding, mechanistic depth, and clinical translation of this field. Most studies follow a linear paradigm of “single modifying enzyme–single substrate–specific phenotype.” While key nodes such as FUT8 and OGT have been successfully identified, this approach fails to systematically resolve the dynamic landscape, internal hierarchy, and complex crosstalk between glycosylation and other modifications - such as phosphorylation and ubiquitination—within the complete glycosylation regulatory network of HCC. At the mechanistic level, research predominantly remains at descriptive correlations. There is insufficient elucidation of the physicochemical mechanisms by which aberrant glycans precisely influence protein conformation, interactions, and subcellular signal transduction. Furthermore, existing technologies struggle to capture the spatiotemporal dynamics of glycosylation in tumor heterogeneity and microenvironmental adaptation (**Figure 6**). Regarding clinical translation, apart from AFP-L3, advancing more glycosylation-based biomarkers or therapeutic targets faces challenges such as standardization of detection, complexity in result interpretation, and the need for large-scale validation. Moreover, drug development directly targeting glycosyltransferases is constrained by low selectivity and potential toxicity. Therefore, future research urgently needs to achieve three major advances: integrating multi-omics and computational biology to map HCC-specific glycosylation regulatory networks and identify core driver nodes; employing structural biology and high-resolution imaging technologies to decipher the precise molecular mechanisms of key modifications; and innovating translational pathways, such as developing immunotherapies targeting specific glycoepitopes, glycosylation-based liquid biopsies, and sensitizing existing treatments by intervening in the glycosylation network.

## 7 Ubiquitin-like modification and HCC

Ubiquitin-like modification is a post-translational protein modification system that regulates diverse biological functions. By covalently conjugating to target proteins, it modulates their stability, localization, and interactions, thereby playing a broad role in the precise regulation of cellular life activities. To date, reported ubiquitin-like proteins include SUMO, URM1, NEDD8, ISG15, FAT10, UFM1, ATG8, ATG12, FUB1, HUB1, and PUP. Their modification processes rely on specific E1 activating enzymes, E2 conjugating enzymes, and E3 ligase cascades, and are dynamically regulated by de-modifying enzymes (72, 73).

### 7.1 SUMOylation and HCC

SUMOylation is the most extensively studied type of ubiquitin-like modification. SUMO proteins were first discovered in 1996, and five SUMO subtypes are currently known: SUMO-1, SUMO-2, SUMO-3, SUMO-4, and SUMO-5 (74). SUMO precursors are cleaved by specific peptidases to expose the C-terminal diglycine motif, and the mature SUMO is then activated by an E1 enzyme in an ATP-dependent manner. Activated SUMO is transferred to the active-site cysteine of an E2 conjugating enzyme, and finally attached to the substrate via an E3 ligase (75). Growing evidence indicates that SUMOylation is involved in multiple processes of HCC (76). Studies have reported significantly elevated expression of SUMO1 in human HCC cell lines and clinical HCC tissue samples compared to non-tumor liver tissues (77). Furthermore, the expression of SAE1 has been found to be significantly upregulated in tumor tissues of HCC patients compared to adjacent non-tumor tissues. SAE1 promotes HCC progression by regulating mTOR signaling through mediating the interaction between SUMO1 and mTOR (78, 79). Interestingly, inhibition of SENP1 activity has been shown to suppress the survival, proliferation, invasion, and migration of HCC cells (80). For example, one study demonstrated that SENP1, as a hypoxia-sensitive gene, is upregulated under hypoxic conditions in solid tumors and promotes tumor proliferation (81). SUMOylation enhances the transcriptional activity of HIF-1α (hypoxia-inducible factor-1α), thereby promoting angiogenesis in hepatocellular carcinoma (82, 83). Additionally, through a SUMO-interacting motif (SIM)-dependent mechanism, SUMOylation significantly enhances the tube-forming capability of vascular endothelial cells *in vitro* and induces migratory and invasive phenotypes in HCC cells *in vitro* (84).

### 7.2 NEDDylation and HCC

NEDDylation is a recently identified type of ubiquitin-like modification. NEDD8, a key ubiquitin-like protein consisting of 76 amino acids (molecular weight ≈ 9.0 kDa), shares 60% homology with ubiquitin and primarily regulates target protein activity through NEDDylation (85). The NEDDylation process conjugates NEDD8 to substrate proteins via a sequential enzymatic cascade. The 81-amino acid precursor encoded by the NEDD8 gene is hydrolyzed by the NEDP1 to expose the C-terminal glycine residue, forming the mature 76-amino acid NEDD8 protein. In an ATP-dependent step, the E1 activating enzyme (NAE) catalyzes the adenylation of the C-terminus of NEDD8, forming a NEDD8–AMP intermediate. The activated NEDD8 is then transferred via a translational reaction to an E2 conjugating enzyme (UBE2M/UBC12 or UBE2F), forming a NEDD8–E2 thioester complex. Finally, an E3 ligase (such as RBX1/RBX2 or MDM2) catalyzes the transfer of NEDD8 from the E2 enzyme to a specific lysine residue on the target protein, forming an isopeptide bond (85, 86). MLN4924, a small-molecule inhibitor of the NEDD8-activating enzyme (NAE), competitively binds to NAE by mimicking the structure of AMP, thereby blocking the transfer of NEDD8 to Cullin proteins. This leads to the inactivation of Cullin–RING ubiquitin ligases (CRLs) and subsequent accumulation of their substrates (e.g., CDT1, p27, Wee1), ultimately inducing DNA damage, cell cycle arrest, and apoptosis (87, 88). Overexpression of NEDDylation pathway components (such as NAE1 and NEDD8) in HCC patients is significantly associated with poor prognosis (89). Future efforts should focus on developing subtype-specific inhibitors and exploring dynamic monitoring technologies based on NEDDylation, such as liquid biopsy for detecting circulating NEDD8 modification levels.

### 7.3 UFMylation and HCC

In 2004, researchers first identified a small protein named UFM1, which shares structural similarities with ubiquitin (90), In 2018, it was officially designated as UFMylation (91). UFM1 primarily exists in the nucleus as an 85-amino acid precursor. The C-terminal glycine residue of UFM1 is exposed through cleavage by UFSP1 or UFSP2, leading to its maturation (92), The mature UFM1 is then activated by binding to the C-terminal glycine of the E1 activating enzyme UBA5, transferred to the E2 conjugating enzyme UFC1 to form a complex (92), and finally conjugated to a lysine residue on the target protein via the E3 ligase UFL1 (93). During this process, UFL1 forms a ternary complex with UFBP1 (UFM1-binding and UFL1 domain-containing protein 1, also known as DDRGK1 or C20orf116) or CDK5RAP3 (CDK5 regulatory subunit-associated protein 3). Both DDRGK1 and CDK5RAP3 are not only substrates of UFMylation but also key regulators of this modification (93–98). After the target protein is modified, UFM1 can be cleaved off by UFSP1 or UFSP2, releasing the UFM1 molecule for reuse in new biological cycles (99, 100). Reported substrates of UFMylation include NLRP3 (101, tau (102, Eg5 (103, MRE11 (104, VCP/p97 (105, PARP1 (106, α-syn (107, PD-1 (108, PD-L1 (109, HERD1 (110, RPL10 (111, p53 (112, DDRGK1 (113, CDK5RAP3 (114, ASC1 (115, RPL26 (116) among others. Through these substrates, UFMylation plays important roles in various biological processes such as endoplasmic reticulum homeostasis, hematopoietic development, DNA damage repair, cell cycle regulation, cell death, and cellular differentiation. Mice with Cdk5rap3 knockout exhibited significant prenatal lethality accompanied by severe liver developmental defects, including delayed cell proliferation and impaired differentiation, highlighting the critical role of CDK5RAP3 in liver development and function. A recent study reported that hepatocyte-specific deletion of *Ufl1* or *Ufbp1* in mice increased susceptibility to high-fat diet (HFD)-induced fatty liver disease and diethylnitrosamine (DEN)-induced HCC. The *Ufl1/Ufbp1* complex acts as a novel upstream repressor of mTOR activation, thereby serving as a defender of liver homeostasis and protecting against liver injury (117). Understanding how these modifications affect the metabolic adaptability of HCC cells may open new research directions for designing therapies targeting metabolic vulnerabilities.

### 7.4 Lactylation and HCC

For a long time, metabolic reprogramming in cancer cells, especially the rewiring of glucose metabolism, has been recognized as a core hallmark. The classical “Warburg effect” describes the tendency of cancer cells to rapidly metabolize glucose via glycolysis, producing substantial lactate even under normoxic conditions (118, 119). This metabolic shift not only provides energy and biosynthetic precursors for rapid proliferation but also leads to significant lactate accumulation in the tumor microenvironment (TME). Historically, lactate was primarily viewed as a metabolic waste product or an energy substrate. However, breakthrough research in recent years has fundamentally altered this understanding by revealing that lactate can act as a precursor for lysine lactylation (Kla), a post-translational modification that covalently modifies lysine residues on both histones and non-histone proteins, thereby directly regulating gene expression and cellular function (120). This discovery has opened a new paradigm for understanding how metabolites can function as signaling molecules to influence epigenetics and cell fate (121). In HCC, the aberrantly active glycolysis provides a rich substrate pool for lactylation. Comprehensive lactylomic profiling has revealed a vast number of lactylation sites in HCC tissues, present not only on histones but also extensively distributed across non-histone proteins (122). For instance, a prospective cohort study of hepatitis B virus-related HCC, integrating lactylomic and proteomic analyses, identified over 9,000 Kla sites, the vast majority of which were located on non-histone proteins. This indicates that lactylation is a widespread modification extending far beyond histones and transcriptional regulation. These modifications preferentially target enzymes involved in key metabolic pathways, such as the tricarboxylic acid (TCA) cycle, and the metabolism of carbohydrates, amino acids, fatty acids, and nucleotides, suggesting a pivotal role for lactylation in regulating cellular metabolic adaptation (123). Therefore, the abnormal accumulation of lactate in HCC not only alters the pH of the microenvironment but also, through this emerging epigenetic-like mechanism, deeply participates in the tumor’s biological processes.

A growing body of evidence demonstrates that lactylation is widely involved in regulating key malignant phenotypes of HCC, including proliferation, metastasis, stemness maintenance, chemoresistance, and immunosuppression. At the level of tumor cells themselves, histone lactylation can directly activate the transcription of oncogenes such as ESM1 and MCM7, thereby promoting cell proliferation, migration, invasion, and epithelial–mesenchymal transition (EMT). Lactylation of non−histone proteins is equally crucial. For instance, lactylation at the K249 site of TPX2 disrupts its binding to protein phosphatase 1, enhancing the phosphorylation of AURKA and driving cell−cycle progression and HCC tumorigenesis. Furthermore, lactylation is a core mechanism underlying acquired therapy resistance in HCC. Studies have shown that histone H3K18la can upregulate the expression of the deubiquitinase USP34, leading to cisplatin resistance (123). Similarly, transcription of HECTD2 is driven by histone lactylation, and its overexpression activates the KEAP1/NRF2 antioxidant pathway, thereby limiting the therapeutic response of HCC to lenvatinib (124). In resistance to hepatic arterial infusion chemotherapy (HAIC), lactylation at the K76 site of IGF2BP3 enhances its binding to m^6^A−modified FSP1 mRNA, upregulates FSP1, and confers resistance to ferroptosis. Together, these findings delineate a complex network through which lactylation drives HCC progression and therapy resistance.

At the tumor−microenvironment level, lactylation serves as a critical bridge in shaping an immunosuppressive milieu. Tumor−derived lactate can upregulate the expression of nuclear protein 1 (NUPR1) in tumor−associated macrophages (TAMs) via histone lactylation, thereby promoting M2−like polarization, increasing the expression of immune−checkpoint molecules such as PD−L1, and leading to CD8^+^ T−cell exhaustion and a diminished response to immunotherapy (125). Similarly, lactate−driven lactylation of SPTAN1 can activate the NOTCH1/HES1 signaling pathway, promoting prostaglandin E2 synthesis, increasing the infiltration of exhausted CD8^+^ T cells, and exacerbating immune suppression (126). These mechanisms partly explain why HCC often presents as an immunologically “cold” tumor and develops resistance to immune−checkpoint inhibitors. Given the central role of lactylation in HCC pathogenesis, prognostic models and diagnostic signatures constructed based on lactylation−related genes have shown promising predictive performance, offering new tools for patient stratification and personalized treatment (127). Therefore, systematically elucidating the regulatory networks, functional impacts, and clinical−translation potential of lactylation in HCC holds significant theoretical and practical importance for developing novel therapeutic strategies and improving patient outcomes (127).

## 8 Discussion and future perspectives

This article systematically reviews the key roles of various classical and emerging PTMs in the pathogenesis, metabolic reprogramming, and therapy resistance of HCC. Integrating existing findings, we propose and elaborate on the conceptual framework of the “PTM code”, aiming to provide a systematic perspective for understanding the complex heterogeneity of HCC and developing novel therapeutic strategies. The concept of the “PTM code” specifically refers to an ordered, dynamic, and biologically informative combinatorial pattern formed by multiple types of PTMs acting on the same protein or protein complex within a specific spatiotemporal context (20). It transcends the isolated study of single modification events, emphasizing the combination and synergistic effects of multiple modifications (such as phosphorylation, acetylation, ubiquitination, glycosylation, etc.). The goal is to decode the complex modification network information into interpretable molecular signatures directly associated with specific biological phenotypes (19).

The uniqueness of this concept lies in its protein specificity and combinatorial determinism. For instance, in HCC, a c-Myc protein simultaneously featuring phosphorylation (activation) at specific sites, acetylation (stabilization), and a lack of ubiquitination (preventing degradation) constitutes a potent “pro-oncogenic code” driving core malignant phenotypes (20). While “PTM crosstalk” emphasizes the mutual influence between two modifications, the “PTM code” focuses more on the overall functional output pattern formed by multiple modifications on a specific protein (128). Compared to the global “PTM network” describing all PTMs and their interactions, the “PTM code” is more functionally oriented, focusing on the modification combinations of individual proteins or complexes. It also differs from the “epigenetic code”, which primarily pertains to histone modifications regulating chromatin states, as its scope is broader, encompassing all types of protein modifications. Therefore, the innovativeness of the “PTM code” lies in its aim to translate the complex, dynamic PTM network information into molecular signatures capable of defining and explaining specific HCC phenotypes (e.g., high-glycolytic, therapy-resistant) (129).

At the systems level, analysis of the “PTM code” requires integrating the synergistic actions of different PTM types on common target proteins, shared signaling pathways, and common biological processes. Taking the tumor suppressor protein p53 as an example, it undergoes phosphorylation, acetylation, ubiquitination, and SUMOylation modifications at multiple sites under different contexts like DNA damage or metabolic stress. The combinatorial pattern of these modifications constitutes a “code” that determines p53’s functional output, precisely regulating whether it triggers cell cycle arrest, apoptosis, or metabolic adaptation (130). Research indicates that specific PTM combinations can recruit distinct “reader” proteins, thereby decoding and executing different biological instructions, providing a molecular basis for understanding p53 dysfunction in HCC (130). At the level of shared signaling pathways, the PI3K/AKT/mTOR pathway is frequently aberrantly activated in HCC. The activity of this pathway is finely regulated by a dynamic “PTM code chain” spanning its components: phosphorylation-mediated activation of upstream receptor tyrosine kinases, interplay between phosphorylation and acetylation of midstream AKT, and ubiquitination regulation of downstream mTOR complex components. Together, these form a dynamic modification network that determines the growth, survival, and metabolic reprogramming phenotypes of HCC cells.

**Figure 7:**
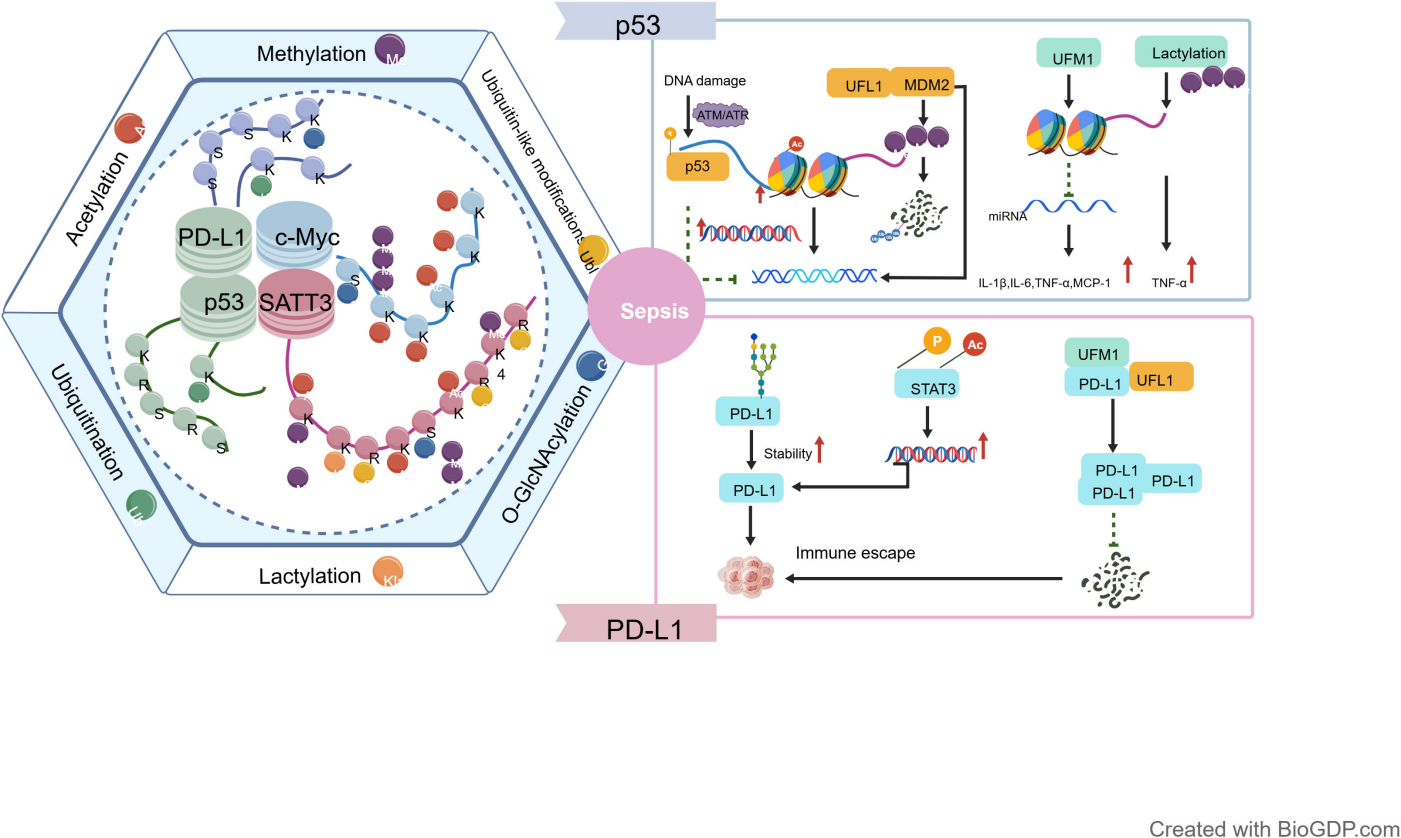
Overview of the PTM regulatory network. The dynamic network of protein post-translational modifications (PTMs) constitutes a complex “PTM code” system. This system precisely regulates the function and fate of key proteins across spatial and temporal dimensions through the combination and cross-talk of multiple modification types, such as phosphorylation, acetylation, ubiquitination, methylation, glycosylation, lactylation, and SUMOylation. Taking the tumor suppressor protein p53 as an example, its activity is intricately regulated by multi-layered PTM codes: DNA damage activates ATM/ATR kinases, triggering phosphorylation at specific serine sites of p53, which further promotes histone acetyltransferase-mediated acetylation at lysine sites, enhancing p53’s transcriptional activity. Meanwhile, ubiquitination mediated by the E3 ubiquitin ligase MDM2 forms a negative feedback loop, precisely controlling p53 protein levels via the proteasomal degradation pathway. This specific combination and sequence of phosphorylation, acetylation, and ubiquitination modifications collectively encode molecular instructions that regulate cell cycle arrest, apoptosis, or senescence. Similarly, within the immune regulatory network, the stability and function of PD-L1 are cooperatively regulated by multiple modifications such as ubiquitination, glycosylation, and phosphorylation, forming a PTM code module that promotes tumor immune evasion. Therefore, the PTM code in hepatocellular carcinoma not only reflects the diversity of modification types but also reveals how the integration of these modifications at key signaling nodes drives malignant tumor phenotypes. This provides a conceptual framework for understanding the pathological mechanisms of HCC at a systems level and for developing precision therapeutic strategies targeting PTM networks.

Furthermore, focusing on a common biological process like “immune evasion”, a “PTM code module” driving the formation of the immunosuppressive tumor microenvironment (TME) in HCC can be constructed. This module integrates modification events at multiple key nodes: for instance, glycosylation of the immune checkpoint protein PD-L1 enhances its stability, promoting tumor cell immune escape; phosphorylation and acetylation of the transcription factor STAT3 act synergistically to activate its transcriptional activity, promoting the expression of immunosuppressive cytokines and PD-L1 (131); simultaneously, relevant inflammatory factors and their signaling pathway proteins are also regulated by various PTMs. These modification events do not exist in isolation but are interwoven, forming a functionally cooperative regulatory network that collectively shapes the immunosuppressive TME of HCC (132). This cross-PTM systems integration model offers a new perspective for holistically deconstructing the complex malignant phenotypes of HCC and developing combined intervention strategies (**Figure 7**).

In the clinical management of HCC, PTM-based biomarkers exhibit different levels of translational maturity. At the highest level of clinical relevance is the fucosylated alpha-fetoprotein subtype (AFP-L3). Studies show that compared to traditional AFP detection, glycosylated AFP has a higher detection rate for early-stage HCC. Its value as a diagnostic and prognostic biomarker for HCC has been validated in multiple studies and is recognized by several clinical guidelines (133). Protein glycosylation is one of the most common and complex PTMs in HCC. Its aberrant changes play a key role in the malignant transformation of HCC by regulating a wide range of pro-tumor signaling pathways and are considered a hallmark feature of HCC (65). Additionally, certain phosphorylated proteins, such as p-ERK, have been shown in tissue microarray studies to correlate with patient prognosis. However, their application in liquid biopsy remains immature, requiring further technological development and validation.

At the stage of clear mechanisms and ongoing clinical translation, specific types of PTM profiles are being validated for their prognostic value in multiple cohort studies. For example, histone acetylation modifications, such as H3K27ac, and emerging lactylation modification profiles show expression patterns in tumor tissue closely linked to HCC progression. Lactylation, a novel PTM mediated by lactate, has emerged as a key regulatory mechanism in the pathological processes of various liver diseases, including HCC (134). Simultaneously, small-molecule tracers targeting the activity of PTM regulatory enzymes (e.g., histone deacetylases HDACs, E3 ubiquitin ligases) are being explored in the field of molecular imaging, aiming for non-invasive, real-time monitoring of enzyme activity to inform precision therapy.

Most emerging PTMs and their complex “code” patterns currently remain in the preclinical potential stage. This includes various acylation modifications such as succinylation and crotonylation. A study utilizing 4D-Label free proteomics combined with PTM peptide enrichment technology systematically profiled nine types of PTMs in HCC tumor tissues, revealing that phosphorylation and ubiquitination were the most frequently altered modifications between tumor and normal tissues, and identified significant crosstalk between PTMs like malonylation-ubiquitination and phosphorylation-ubiquitination. Another study conducted a comprehensive analysis of nine PTMs in paired tissues from HCC patients using liquid chromatography-tandem mass spectrometry, identifying over 60,000 modification sites, further confirming the widespread existence of these emerging PTMs. However, the specificity of these modifications as biomarkers, their detectability in body fluids, and their stable association with clinical outcomes still require large-scale technological development and rigorous preclinical validation.

Therapeutic strategies targeting PTMs in HCC present a clear translational ladder. Among drugs already on the market or in advanced clinical stages, histone deacetylase inhibitors (HDACis) like panobinostat have been approved for other hematologic malignancies. However, in clinical trials for HCC, HDACis have shown limited single-agent activity, suggesting the need for future exploration of combination strategies with other therapies to enhance efficacy [1]. Similarly, ubiquitin-proteasome inhibitors (e.g., bortezomib) have shown poor efficacy in HCC, highlighting the importance of targeting specific molecules in the HCC context rather than broadly inhibiting the entire pathway.

Current hotspots in early clinical and preclinical research focus on more specific targets. For example, small-molecule drugs targeting specific E3 ubiquitin ligases (e.g., MDM2 inhibitors) or deubiquitinating enzymes (DUBs) are under active development. Growing evidence indicates that dysfunction of many DUBs is associated with tumorigenesis, making DUBs a new therapeutic target for HCC (135). Furthermore, compounds modulating O-GlcNAcylation show potential in preclinical models for overcoming therapy resistance. O-GlcNAcylation is a dynamic PTM whose dysregulation participates in HCC malignant progression and drug resistance through various molecular signaling pathways. Multi-target combination strategies aimed at complex “PTM codes”, such as HDACis combined with kinase inhibitors, represent an important current research direction, aiming to overcome compensatory mechanisms of single pathways through synergistic effects.

However, translating PTM-based strategies into clinical practice faces a series of core challenges. First, the high dynamism and tissue specificity of PTMs increase the complexity of targeted therapy. Second, targeting modifier enzymes (e.g., “writers” or “erasers”) may cause systemic toxicity since the same enzyme may regulate multiple substrate proteins. Third, the lack of delivery systems capable of precisely delivering therapeutic agents to the tumor site limits drug efficacy and amplifies side effects. Perhaps the greatest challenge lies in translating complex “PTM code” information into actionable clinical decisions. This requires integrating high-throughput proteomics, computational biology, and medicinal chemistry to develop systematic approaches capable of parsing PTM networks, predicting therapeutic responses, and designing more precise intervention tools. In the future, integrating multi-omics data and artificial intelligence models holds promise for deciphering PTM regulatory networks, thereby advancing the establishment of a personalized classification and treatment framework for HCC.
